# GC-MLP: Graph Convolution MLP for Point Cloud Analysis

**DOI:** 10.3390/s22239488

**Published:** 2022-12-05

**Authors:** Yong Wang, Guohua Geng, Pengbo Zhou, Qi Zhang, Zhan Li, Ruihang Feng

**Affiliations:** 1 School of Information Science and Technology, Northwest University, Xi’an 710127, China; 2 School of Arts and Communication, Beijing Normal University, Beijing 100875, China

**Keywords:** 3D point cloud, neural network, shape analysis, local aggregation operation, graph convolution multilayer perceptron

## Abstract

With the objective of addressing the problem of the fixed convolutional kernel of a standard convolution neural network and the isotropy of features making 3D point cloud data ineffective in feature learning, this paper proposes a point cloud processing method based on graph convolution multilayer perceptron, named GC-MLP. Unlike traditional local aggregation operations, the algorithm generates an adaptive kernel through the dynamic learning features of points, so that it can dynamically adapt to the structure of the object, i.e., the algorithm first adaptively assigns different weights to adjacent points according to the different relationships between the different points captured. Furthermore, local information interaction is then performed with the convolutional layers through a weight-sharing multilayer perceptron. Experimental results show that, under different task benchmark datasets (including ModelNet40 dataset, ShapeNet Part dataset, S3DIS dataset), our proposed algorithm achieves state-of-the-art for both point cloud classification and segmentation tasks.

## 1. Introduction

With the substantial improvement in the performance of 3D scanning equipment, the application scenarios of 3D data in computer vision are also increasing, and include remote sensing mission, autonomous driving, robotics. In contrast with the two-dimensional regularly arranged images, the three-dimensional point cloud data are a collection of points in space which are characterized by being sparse, irregular and disordered. Therefore, means of effectively and quickly improving the performance of traditional optical remote sensing images in road segmentation, three-dimensional urban modeling, forestry monitoring and other tasks in this field through the point cloud approach to the original sensor data characteristics have attracted extensive attention.

To solve these problems, some scholars have carried out a lot of research, which can be roughly divided into three aspects: multi-view, voxel and point. The multi-view-based method [1,2] mainly projects three-dimensional data onto regular two-dimensional images, and then performs two-dimensional convolution operations. However, there is still a certain loss of spatial information in the process of projection. The early works on the Voxel-based method [3,4,5]: mainly quantified irregular three-dimensional data to form regular three-dimensional data, and then applied three-dimensional convolution to extract features. However, the quantization operation of this method greatly increases the computational cost. The point-based methods [6,7,8] directly takes a sparse or dense point set as input, and then uses a multilayer perceptron (MLP) to extract features.

Recently, some scholars directly performed convolution operations on irregular point cloud data by designing convolution kernels [9,10]. These methods mainly distribute convolution kernel weights for local adjacent points, make local point cloud data regularized and then use convolution operations. In addition, some research works established the topological relationship between points through the graph structure [11,12,13], and then performed convolution operations. However, in the complex point cloud density environment, different adjacent points have different feature correspondences, so the fixed/isotropic convolution kernel will make the final effect poor.

In this paper, we propose an adaptive weighted graph convolutional multilayer perceptron, namely GC-MLP. The main contributions of this paper can be summarized as follows:

(a) We propose a point cloud processing method based on adaptive weight graph convolution multilayer perceptron. The adaptive weight method greatly improves the flexibility of the algorithm by defining convolution filters at any relative position, and then calculating the aggregate weights on all adjacent points.
(b) We propose a convolution multilayer perceptron, which improves the generalization performance through convolutional sparse links, and improves the model capacity through MLP’s dynamic weights and global receptive field. The experimental results show that our proposed algorithm achieves the state of the art on both classification and segmentation benchmark datasets.

## 2. Related Work

For 2D image understanding, pixels are arranged in a regular grid and can be processed using methods such as convolution. In contrast, the 3D point cloud is disordered and irregular in 3D space, and the essence of point cloud is collection. At this stage, methods based on learning 3D point cloud processing are mainly divided into the following types: projection-based, voxel-based and point-based networks.

### 2.1. Projection-Based Networks

Due to the success of 2D image processing in deep learning, early works [1,2,14] first projected point clouds onto 2D images through multiple views, and then used traditional convolutions for feature learning. However, point cloud data have irregularities in three-dimensional space, and two-dimensional images are regular, so the process of converting three-dimensional images into two-dimensional images results in some data loss. In a recent studies, Tatarchenko et al. [15] proposed a surface convolution. The deep convolutional network structure first projects the local surface geometry of each point onto the tangent planes around the point, and then convolves the sliced image. Lin et al. [16] proposed a flattened convolution method for point cloud structure. This method first maps the local point cloud to a two-dimensional plane through interpolation, and then uses traditional convolutions to extract features. Lang et al. [17] proposed a new encoder PointPillars. The method firstly divides the 3D point cloud on the x–y plane to form a uniform point cloud column, converts it into a sparse virtual image through a learnable encoder and finally performs a convolution operation on it to extract the features. To reduce the required computing resources, Li et al. [18] proposed a transformation function, which aggregates point clouds into 2D image space before using affine transformation for transformation. Then, the convolution algorithm is applied to the transformed image space.

### 2.2. Voxel-Based Networks

Voxel-based methods convert irregular 3D point cloud data into regular 3D data [19,20], and then perform 3D convolution on them to extract features. Due to the loss of spatial information in the conversion process, and with the increase in voxel resolution, the memory and calculation amount also greatly increase. For such problems, recent studies have used sparse structures to obtain smaller grids with better performance. For example, the OctNet [21] and Kd-Net [22] networks divide point clouds by building trees. However, due to the quantization of the voxel grid, there is still a certain loss of information. Park et al. [23] have effectively improved the computing efficiency by using lightweight self-attention to encode the voxel hash architecture.

### 2.3. Point-Based Networks

Unlike projections in 2D and voxel quantization in 3D to regularize the data, this section discusses the direct input of point cloud data as features into deep networks [24,25,26,27,28]. PointNet [29] first proposed to use MLP for feature extraction independently for each point, and then aggregate the global features through a symmetric function (global max pooling). However, due to the lack of local features captured in this structure, the ability to recognize fine-grained categories and generalization in complex scenes is limited. Recent studies [30,31,32] have proposed more ways of capturing local features, such as multi-scale features and weight features. Among them, Qi et al. [33] iteratively extracted local features from the spatial point cloud by calculating the spatial distance, which effectively solved the problem of local adjustment loss in the PointNet structure network. To better capture the characteristics of different scales, Wang et al. [34] proposed a multi-scale fusion module, which can adaptively consider the information of different scales and establish fast descent branches to bring more abundant gradient information.

Recently, some local feature learning methods that directly input point cloud data into continuous convolutions have also yielded good experimental results. PointCNN [35] transforms the local input point cloud data into regularized data through X-conv transformation, and then performs convolution operation. SpiderCNN [36] extends the convolution operation from a regular grid to an embeddable irregular point set through a defined convolution filter, and captures local informative features through the filter. KPConv [9] captures local information through the local radius search and extracts features through flexible Kernel convolution with an arbitrary number of points. RS-CNN [37] first performs random sampling to establish a neighborhood set of sampling points, and then calculates the distance between the neighbor point and the center point in each neighborhood to bring the local prior relationship into the convolution operation. ShellNet [38] divides the neighbors of sampling points into different shells, then extracts features from the points of the same shell, and finally aggregates the local features of different shells. Qiu et al. [39] proposed a geometric back-projection network for point cloud classification, which uses the idea of error correction feedback structure to fully capture the local features of point clouds.

Some studies treat the set of points in the point cloud as the vertices of the graph in the graph network [40,41,42], and generate directed edges of the graph based on the neighborhood of this point. DGCNN [12] captures local information through EdgeConv, and updates the local information at each layer through the nearest neighbor number. However, EdgeConv only considers the distance between the coordinates of the point and each neighbor point, ignoring the vector direction of the adjacent points, and loses part of the local geometric information. Wang et al. [11] proposed a graph attention convolution, which dynamically distributes the attention weights of neighboring points by combining spatial locations and specific attributes. ECC [43] proposes a local convolution of graphs, where the weights of the convolution kernels depend on the values of adjacent edges and can be dynamically updated for each specific input. SPG [44] captures contextual relationships by building a super-dot graph structure, and then performs convolutional learning. Landrieu et al. [45] proposed a graph-structured contrast loss, which is combined with a cross-partition weighting strategy to generate point embeddings with high contrast at object boundaries. Lin et al. [13] proposed a 3D graph convolutional network for processing 3D point cloud data. The shape and weight of the convolution kernel are obtained during training, and graph max pooling captures features of different scales. Yang et al. [46] proposed a continuous CRF graph convolution (CRFConv) to construct an encoder–decoder network, in which the CRFConv embedded in the decoding layer can recover the details of high-level features lost in the encoding phase to enhance the network’s positioning capability.

## 3. Methods

In this section, we first review the overall idea of direct point processing (Section 3.1), and then we go on to introduce adaptive weighted graph convolutional multilayer perceptrons (AWConvMLP) (Section 3.2). Finally, we introduce the transformation process of convolutional multilayer perceptrons (ConvMLP) (Section 3.3).

### 3.1. Overview

Dataset: For point cloud set $P={\left\{p_{i}\right\}}_{i=1}^{N}$ with *N* points and its corresponding point cloud feature $F={\left\{f_{i}\right\}}_{i=1}^{N}$, where $p_{i}\in R^{\chi }$ is the point cloud coordinate and color information of the *i*-th point, and $f_{i}\in R^{C}$ is its corresponding point cloud feature.

General formulation: When the point cloud set is input, the local aggregation network first calculates the relative position $\Delta p_{ij}\left(\Delta p_{ij}=p_{i}-p_{j}\right)$ of any point $p_{i}$ and the feature $f_{i}$ of its adjacent points. Then, converts the local adjacent point features into a new feature map through the conversion function G(•,•). Finally, global features are extracted using channel symmetric aggregation functions.

(1) $$f_{i}^{\prime }=S\left(G\left(\Delta p_{ij},f_{i}\right)|j\in N\left(i\right)\right)$$

where N(i) represents the nearest neighbor of point $p_{i}$. Symmetric aggregate function *S* is usually the maximum, average or sum.

### 3.2. Adaptive Weight Graph Convolution Multilayer Perceptron

We define a point *P* and a set of adjacent spatial points to form a graph structure G(V,E), where V={1,2,⋯,N} and $E\subseteq \left|V\right|\times \left|V\right|$ represent sets of vertices and edges, respectively. Definition N(i)={j:(i:j)∈E}∪{i} (including itself) is the number of neighbors of point $p_{i}$.

**Figure 1:**
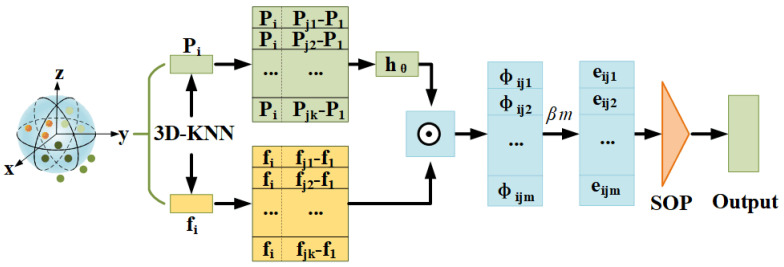
Processing flow of AWConvMLP. Firstly, the local aggregation network extracts the features of target point P, convolutes its relative position, then calculates the aggregation weight of all dimensional convolution kernels and finally extracts the feature by channel symmetric aggregation function (Max, average or sum. Here, max pooling is used [12,42].

First, we design a convolutional filter at arbitrary relative positions such that it focuses on the most relevant parts of the neighborhood for learning (As shown in Figure 1), enabling the convolutional kernel to dynamically adapt to the object’s structure.

(2) $$\Psi_{ijm}=h_{\theta }\left(\Delta p_{ij}\right)$$

where $\Psi_{ijm}$ represents the weight of M filters and $h_{\theta }$ is the feature mapping function. Here, we use MLP, whose dimension is from dim to 2*dim and back to dim. $\Delta p_{ij}=\left[p_{i},p_{j}-p_{i}\right]$ is the graph vertex and its relative position, [•,•], is the concatenation operation.

In addition, the aggregation weight corresponding to each adjacent point convolution kernel is as follows:

(3) $$\begin{array}{cc} G(\Delta p_{ij},f_{i}) & =e_{ijm}\\ \; & =\beta_{m}\langle \Psi_{ijm},f_{i}\rangle \end{array}$$

where $\beta_{m}$ represents the point-wise MLP. $\langle •,•\rangle$ is the inner product of two vectors. $f_{i}$ is correspondingly defined as $\left[f_{i},f_{j}-f_{i}\right]$.

Finally, we define the output features of point $p_{i}$ by applying an aggregate function to all edge features in the neighborhood:

(4) $$f_{i}^{\prime }=s_{m}\left(e_{ijm}\right)$$

where $s_{m}$ represents the max pooling function.

### 3.3. Convolution Multilayer Perceptron

For the traditional convolution with a fixed convolution kernel, it contains local connections, which has the advantages of high computational efficiency, but has the characteristics of a poor effect in complex environments. Although the point-wise MLP structure has the characteristics of large model capacity, the local generalization ability needs to be improved.

In view of the advantages and disadvantages of the two, we propose a model ConvMLP, which improves the generalization performance through sparse links, and improves the model capacity through dynamic weights and point-wise MLP.

First, the characteristic matrix output in the previous stage is used as the input data of the model, the graph structure matrix is obtained through the graph network, then it is input into a convolution residual multilayer perceptron structure, and finally, the graph convolution multilayer perceptron feature with maximum pooling is output.

(5) $$\epsilon_{ijm}^{\prime }=\rho \left(conv\left(g\left(p,f\right)\right)\right)$$

(6) $$f_{i}^{\prime }=S_{m}\left(\zeta \left(Norm\left(\epsilon_{ijm}^{\prime }\right)\right)\right)$$

where g(•,•) represents the graph network, $g=[\Delta p_{ij},f_{i}]$, conv denotes the convolution operation, ρ stands for the activation function, $\epsilon_{ijm}^{\prime }$ represents the *j*-th feature vector after convolution, Norm represents a normalized function, ζ represents channel MLP, and $f_{i}^{\prime }$ represents the feature vector output through the ConvMLP layer.

## 4. Experimental Results and Analysis

We evaluate our model on three different datasets for point cloud classification, part segmentation and semantic scene segmentation. For classification, we use the benchmark ModelNet40 dataset [47]. For object part segmentation, we use the ShapeNet Part dataset [48]. For semantic scene segmentation, we use the Stanford Large 3D Indoor Space (S3DIS) dataset [49].

### 4.1. Classification

**Data:** We train and evaluate the proposed classification task model on the ModelNet40 dataset and compare the results with those of previous work. ModelNet40 is a synthetic dataset consisting of computer-aided design (CAD) models, containing 12,311 CAD models given as triangular meshes, among which 9843 samples are used for training and 2468 samples are used for testing. For evaluation metrics, we use the mean accuracy within each category (mAcc) and overall accuracy (OA) across all categories.

**Figure 2:**
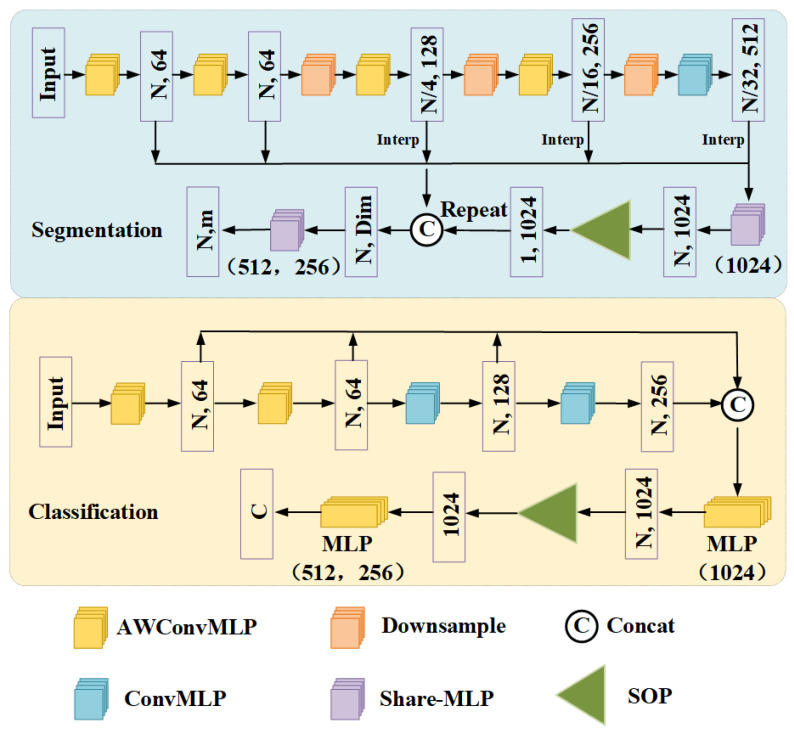
The GC-MLP network structure is used to classify and divide tasks. The convolution of the convolution layer is a standard convolution operation. The first two layers of the segmentation model use normal AWconvMLP, the last three layers use downsampling and AWconvMLP structures, and finally use linear interpolation and skip link connection methods to upsample and aggregate the features of each layer. Among them, Dim represents 2112 dimensions (2048+64 (category vector)) in partial segmentation, Dim represents 2048 dimensions in semantic segmentation and the classification model uses dynamic structure.

**Network configuration:** The network structure flow of the classification task is shown in Figure 2. GC-MLP replaces the first two layers and the last two layers of the four-layer EdgeConv in the DGCNN algorithm with AWConvMLP and ConvMLP, respectively. The number of nearest neighbors k for each layer is set to 20. Shortcut connections employ a shared fully connected layer (1024) to aggregate multi-scale global features, where the global features of each layer are obtained through a max pooling function. The initial learning rate of the SGD optimizer is 0.03 and the momentum is 0.9. The normalization and activation functions of each layer are batch normalization and LeakyReLU, respectively. The dropout rate is set to 0.5. The batch size of all trained models is set to 32 and trained for 250 epochs. We use PyTorch [50] to implement and train the network on a RTX 3090 GPU. For other tasks, hyperparameters are chosen in a similar manner.

**Results:** To make the comparison clearer, we additionally list the input data type and number of points corresponding to each model algorithm. The experimental results of the classification task are shown in Table 1. As can be seen from Table 1, compared with recent model algorithms, our proposed model has state-of-the-art performance results under the same dataset. Compared with the CSANet algorithm, our algorithm achieves 1.1% and 0.6% improvement in mAcc and OA, respectively. Compared with the MFNet algorithm, our algorithm is slightly lower in mAcc, but has 0.3% improvement in OA. Compared with the DGCNN model algorithm, it improves by 0.8% in mAcc and 0.5% in OA.

### 4.2. Part Segmentation

**Data:** We train and evaluate the proposed part segmentation task model on the ShapeNet dataset and compare the results with those of previous work. The dataset has 16 categories, and there are 2–6 types of 50 shapes in each category (2048 points are sampled for each shape), and a total of 16,881 3D models in all categories (including 14,007 in the training set and 2874 in the test set). For the evaluation metrics, we use the mean class IoU (mcIoU) per class and the mean instance IoU (mIoU) per instance.

**Network configuration:** The network structure flow of some segmentation tasks is shown in Figure 2. We replace the EdgeConv layer in the DGCNN algorithm with AWConvMLP, transition downsample, and ConvMLP. Experimental settings such as SGD, normalization, activation function, and dropout rate are consistent with the classification tasks.

**Figure 3:**
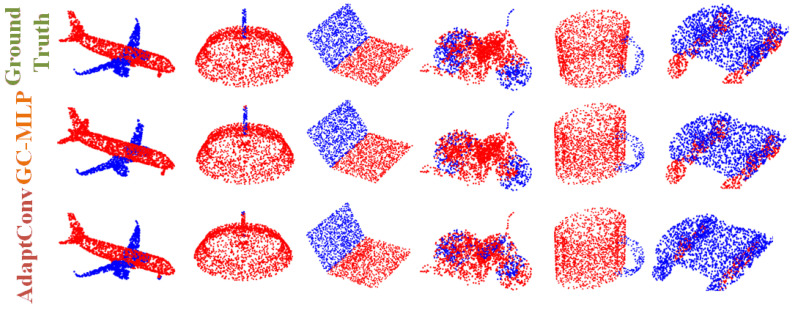
Visualization of shape part segmentation results on ShapeNet Parts. The first row is the ground truth, and the second row is the predictions of our GC-MLP. From left to right are the airplane, chair, earphone, lamp, laptop, motorbike, mug and skateboard.

**Figure 4:**
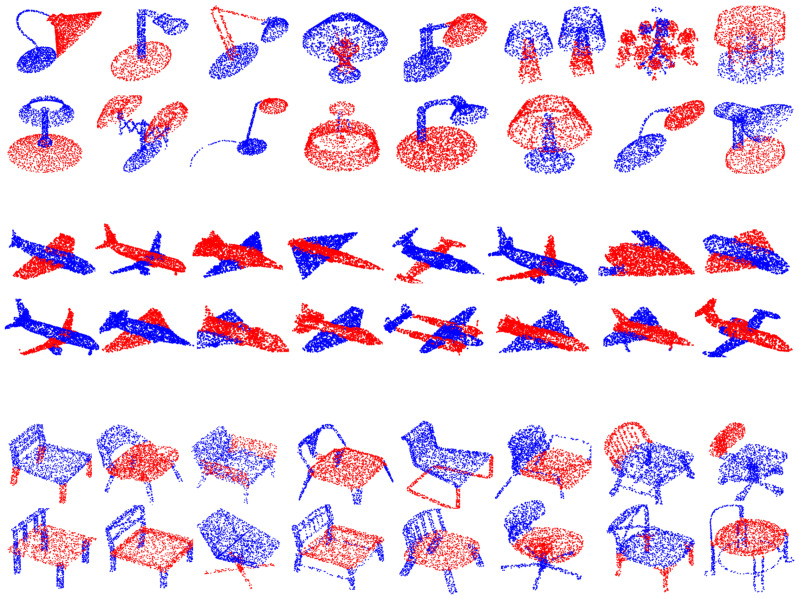
Visualization of the part segmentation results for the lamps, airplanes and chairs.

**Results:** The experimental results of some segmentation tasks are shown in Table 2. The visualization results of part segmentation are shown in Figure 3, and more visualization results are shown in Figure 4. As can be seen from Table 2, compared with recent model algorithms, our proposed model has state-of-the-art performance results on the same dataset. As can be seen from Figure 3, the part segmentation prediction of GC-MLP is clear and close to the ground truth. Compared with the DGCNN model algorithm, it improves by 1.3% in mcIoU and 0.9% in mIoU.

### 4.3. Indoor Scene Segmentation

**Data:** We evaluate the proposed semantic segmentation model on the real-world point cloud semantic segmentation S3DIS dataset and compare the results with those of previous work. The dataset contains 6 indoor areas (between 23 and 68 rooms in each area), 11 scenes (e.g., office, meeting room, open space) and 13 sub-categories (e.g., ceiling, wall, chair) for a total of 272 areas. For evaluation metrics, we use the mean class-wise intersection over union (mIoU) per class.

**Network configuration:** We follow the same setting as prior study [9], where each point is represented as a 6D vector (XYZ, RGB). We performed six cross-validations with six regions, each with five regions for training and one region for validation. Due to the overlap between the other regions except Region 5, we will report the metrics in Region 5 separately.

**Figure 5:**
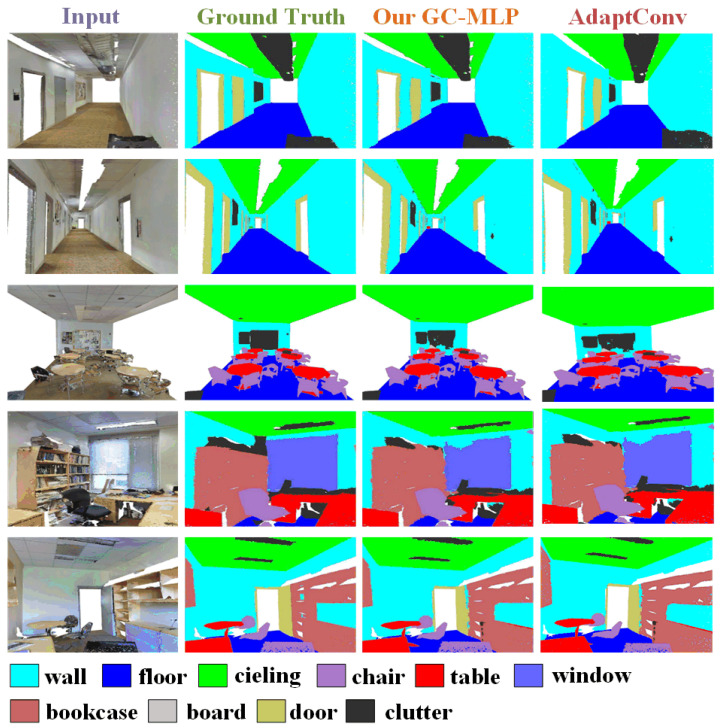
Visualization of semantic segmentation results on S3DIS Area-5. The first column shows original scene inputs, the second column shows the ground truth annotations, and the third column shows the scenes segmented by our proposed model GC-MLP.

**Results:** The experimental results of the semantic segmentation task are shown in Table 3. The visualization results of the semantic segmentation are shown in Figure 5. As can be seen from Table 3, compared with recent model algorithms, our proposed model obtains state-of-the-art performance results on the same dataset. As can be seen from Figure 5, the semantic segmentation prediction of GC-MLP is very close to the ground truth. Compared with the DGCNN model algorithm, it improves by 1.3% in mIoU.

### 4.4. Ablation Studies

In order to verify some choices of the structure in this paper, we conducted comparative experiments with different graph convolution structures based on the ModelNet40 dataset, and the results are shown in Table 4.

AWConvMLP and ConvMLP: On the premise of keeping other parameters the same and only replacing the model, this paper discusses and verifies the proposed model algorithm with standard graph convolution (DGCNN), ConvMLP and AWConvMLP. The results are shown in Table 4.

It can be seen from Table 4 that compared with other model algorithms, the model algorithm (GC-MLP) combining AWConvMLP and ConvMLP has better experimental results.

AWConvMLP VS ConvMLP: Inspired by channel MLP, we add MLP on the basis of graph convolution, and test the first two layers and the last two layers of convolution and MLP combined experiments, the results of which are shown in Table 4.

It can be seen from Table 4 that when the convolution is combined with the MLP, experimental results have better improvement. This is due to the local information interaction between the MLP and the convolution layer, which makes up for a certain loss of spatial information.

### 4.5. Robustness Test

To evaluate the robustness of the proposed model, we conducted test experiments on point cloud density and nearest neighbors on the ModelNet40 dataset.

**Figure 6:**
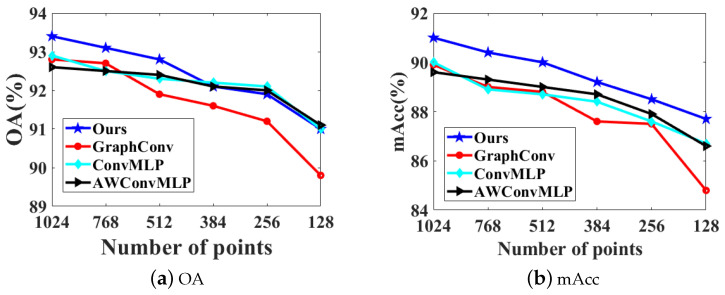
Robustness test on ModelNet40 for classification. GraphConv indicates the standard graph convolution network. ConvMLP indicates that we replace the ablation of the AWConvMLP layer in GC-MLP with ConvMLP. AWConvMLP indicates that we replace the ablation of the ConvMLP layer in GC-MLP with AWConvMLP.

For the point cloud density experiment: under the premise of unified parameter settings, this paper randomly selects a series of points from 128 to 1024, and conducts a comparative test experiment with other graph convolution structures. The results are shown in Figure 6.

It can be seen from Figure 6 that, compared with other graph convolution structure models, the model proposed in this paper has better robustness in point cloud density experiments.

For the nearest neighbor number experiment, under the premise of unified parameter settings, this paper randomly selects a series of representative nearest neighbor numbers from 5 to 40, and conducts a comparative test experiment under the number of 1024 points. The results are shown in Table 5.

It can be seen from Table 5 that when the number of nearest neighbors k = 20, it has better performance. As the number of nearest neighbors decreases within a certain range, the visual receptive field is also limited, and the performance also decreases. However, when the number of nearest neighbors reaches a certain level, as the number of nearest neighbors increases, nearby local information will also be incorporated, and the performance will also decrease.

### 4.6. Efficiency

In order to calculate the computational complexity of the proposed model structure, this paper calculates the parameters and the performance achieved by the model based on the classification task of the ModelNet40 dataset. The parameters and model performance of the proposed model and excellent algorithms in recent years are shown in Table 6.

It can be seen from Table 6 that compared with other model structures, the model proposed in this paper not only has less model parameters, but also has a state-of-the-art performance in classification tasks.

## 5. Conclusions

In this paper, we propose a method based on the graph convolution multilayer perceptron method for the problem of 3D point cloud feature learning. Experimental results show that the proposed algorithm not only achieves good experimental performance in point cloud classification and segmentation tasks under the benchmark dataset, but also has good robustness and efficiency. Among them, AWConvMLP enables the convolution kernel at any relative position to dynamically adapt to the structure of the object by learning the features of the most relevant parts of the neighborhood, which effectively improves the flexibility of point cloud convolution. The global feature learning is further carried out through the weight-sharing multi-layer perceptron. ConvMLP improves the generalization performance through sparse links, and improves the model capacity through dynamic weights and global receptive fields.

## Figures and Tables

**Table 1:** Classification results on ModelNet40 dataset.

Method	Input	Points	mAcc (%)	OA (%)
3DShapeNetParts [47]	voxel	-	77.3	84.7
VoxNet [5]	voxel	-	83.0	85.9
Subvolume [51]	voxel	-	86.0	89.2
PointNet [29]	xyz	1k	86.0	89.2
PointNet++ [33]	xyz, normel	5k	-	91.9
Kd-Net [22]	xyz	1k	-	90.6
SpecGCN [52]	xyz	1k	-	92.1
SpiderCNN [36]	xyz, normel	5k	-	92.4
PointCNN [35]	xyz	1k	88.1	92.2
SO-Net [30]	xyz, normel	5k	-	93.4
DGCNN [12]	xyz	1k	90.2	92.9
KPConv [9]	xyz	1k	-	92.9
3D-GCN [13]	xyz	1k	-	92.1
PointASNL [28]	xyz, normel	6.8k	-	93.2
AdaptConv [42]	xyz	1k	90.7	93.4
DRNet [10]	xyz	1k	-	93.1
CSANet [34]	xyz	1k	89.9	92.8
MFNet [53]	xyz	1k	91.4	93.1
ours	xyz	1k	91.0	93.4

**Table 2:** Part segmentation results on the ShapeNetPart dataset evaluated as the mean class IoU (mcIoU(%)) and mean instance IoU (mIoU(%)).

Method	mcIoU	mIoU	Air Plane	Bag	Cap	Car	Chair	Ear Phone	Guitar	Knife	Lamp	Laptop	Motor	Mug	Pistol	Rocket	State	Table
KdNet [22]	77.4	82.3	80.1	74.6	74.3	70.3	88.6	73.5	90.2	87.2	81	94.9	87.4	86.7	78.1	51.8	69.3	80.3
PointNet [29]	80.4	83.7	83.4	78.7	82.5	74.9	89.6	73	91.5	85.9	80.8	95.3	65.2	93	81.2	57.9	72.8	80.6
PointNet++ [33]	81.9	85.1	82.4	79	87.7	77.3	90.8	71.8	91	85.9	83.7	95.3	71.6	94.1	81.3	58.7	76.4	82.6
SO-Net [30]	81	84.9	82.8	77.8	88	77.3	90.6	73.5	90.7	83.9	82.8	94.8	69.1	94.2	80.9	53.1	72.9	83
DGCNN [12]	82.3	85.2	84	83.4	86.7	77.8	90.6	74.7	91.2	87.5	82.8	95.7	66.3	94.9	81.1	63.5	74.5	82.6
PointCNN [35]	-	86.1	84.1	86.4	86	80.8	90.6	79.7	92.3	88.4	85.3	96.1	77.2	95.3	84.2	64.2	80	83
PointASNL [28]	-	86.1	84.1	84.7	87.9	79.7	92.2	73.7	91	87.2	84.2	95.8	74.4	95.2	81	63	76.3	83.2
3DGCN [13]	82.1	85.1	83.1	84	86.6	77.5	90.3	74.1	90.9	86.4	83.8	95.6	66.8	94.8	81.3	59.6	75.7	82.8
AdaptConv [42]	83.4	86.4	84.8	81.2	85.7	79.7	91.2	80.9	91.9	88.6	84.8	96.2	70.7	94.9	82.3	61	75.9	84.2
CRFConv [46]	83.5	85.5	83.9	84.8	83.0	80.2	91.8	77.9	91.8	86.9	84.9	95.6	77.8	95.6	82.0	64.4	75.3	80.8
ours	83.6	86.1	84.8	84.8	86.2	79.5	91.4	78.7	91.2	87.8	85.5	96.1	73.6	94.8	83.4	60.8	77.3	83

**Table 3:** Semantic segmentation results on S3DIS dataset evaluated on Area 5. We report the mean classwise IoU (mIoU(%)).

Method	mIoU	Ceiling	Floor	Wall	Beam	Column	Window	Door	Table	Chair	Sofa	Book-Case	Board	Clutter
PointNet [29]	41.1	88.8	97.3	69.8	0.1	3.9	46.3	10.8	59	52.6	5.9	40.3	26.4	33.2
SegCloud [54]	48.9	90.1	96.1	69.9	0	18.4	38.4	23.1	70.4	75.9	40.9	58.4	13	41.6
PointCNN [35]	57.3	92.3	98.2	79.4	0	17.6	22.8	62.1	74.4	80.6	31.7	66.7	62.1	56.7
PCCN [55]	58.3	92.3	96.2	75.9	0.3	6	69.5	63.5	66.9	65.6	47.3	68.9	59.1	46.2
PointWeb [25]	60.3	92	98.5	79.4	0	21.1	59.7	64.8	76.3	88.3	46.9	69.3	64.9	52.5
HPEIN [8]	61.9	91.5	98.2	81.4	0	23.3	65.3	40	75.5	87.7	58.5	67.8	65.6	49.4
GAC [11]	62.8	92.2	98.2	81.9	0	20.3	59	40.8	78.5	85.8	61.7	70.7	74.6	52.8
PointASNL [28]	62.6	94.3	98.4	79.1	0	26.7	55.2	66.2	83.3	86.8	47.6	68.3	56.4	52.1
AdaptConv [42]	67.9	93.9	98.4	82.2	0	23.9	59.1	71.3	91.5	81.2	75.5	74.9	72.1	58.6
CRFConv [46]	66.2	93.3	96.3	82.2	0	23.7	60.3	68.2	82.4	86.0	63.4	73.8	72.4	58.9
ours	67.2	95.2	98.5	82.7	0	20.4	58.7	70	91.2	81.7	76.5	66.8	68.8	62.2

**Table 4:** Comparison of classification results of different schemes based on ModelNet40 dataset.

GraphConv	AWConvMLP	ConvMLP	GC-MLP						mAcc (%)	OA (%)
			First Two Layers			Last Two Layers				
			Non	MLP	ResMLP	Non	MLP	ResMLP		
√									89.9	92.8
	√								89.6	92.6
		√							90.0	92.9
			√					√	90.2	92.8
				√				√	91.0	93.4
					√			√	90.3	93.2
				√		√			90.4	93.2

**Table 5:** Results of our classification network with different numbers k of nearest neighbors.

K	mAcc (%)	OA (%)
5	88.1	92.2
10	90.4	93.0
20	91.0	93.4
40	90.7	93.2

**Table 6:** The number of parameters and overall accuracy of different models.

Method	Parameters	mAcc(%)	OA(%)
PointNet [29]	3.5 M	86.0	89.2
PointNet++ [33]	1.48 M	-	91.9
DGCNN [12]	1.81 M	90.2	92.9
KPConv [9]	14.3 M	-	92.9
PosPool [56]	19.4 M	-	93.2
AdaptConv [42]	1.85 M	90.7	93.4
ours	2.22 M	91.0	93.4

## Data Availability

Restrictions apply to the availability of these data. The data were obtained from Stanford University and accessed on 2 November 2021, available at https://goo.gl/forms/4SoGp4KtH1jfRqEj2/ with the permission of Stanford University. Publicly available datasets were analyzed in this study. The datasets was accessed on 2 November 2021. It can be found here: http://modelnet.cs.princeton.edu/ and https://www.shapenet.org/.
